# Anti-CGRP monoclonal antibody therapy for migraine is not associated with early adverse bone effects: a prospective, observational, controlled, cohort study

**DOI:** 10.3389/fneur.2026.1851653

**Published:** 2026-05-29

**Authors:** Giulia Mallucci, Chiara Camponovo, Alberto Cordella, Rosaria Sacco, Alessia Bellotti, Alessandro Ceschi, Pierpaolo Trimboli, Claudio Gobbi, Chiara Zecca

**Affiliations:** 1 Neurocenter of Southern Switzerland, EOC, Lugano, Switzerland; 2 Ente Ospedaliero Cantonale, Regional Hospital of Lugano, Clinic for Endocrinology and Diabetology, Lugano, Switzerland; 3 Faculty of Biomedical Sciences, Università della Svizzera Italiana, Lugano, Switzerland; 4 Clinical Trial Unit, Ente Ospedaliero Cantonale, Lugano, Switzerland; 5 Division of Clinical Pharmacology and Toxicology, Institute of Pharmacological Sciences of Southern Switzerland, Lugano, Switzerland

**Keywords:** bone mineral density, bone turnover markers, calcitonin gene-related peptide, migraine, monoclonal antibodies

## Abstract

**Background and objective:**

Calcitonin gene–related peptide (CGRP) is a key mediator in migraine and a target of recent preventive therapies. Since CGRP is also expressed in bone and involved in skeletal regulation, concerns have been raised regarding potential bone effects of CGRP pathway inhibition. We report a prespecified 6-month analysis of a prospective, observational controlled, cohort study evaluating early bone outcomes during anti-CGRP monoclonal antibody (mAb) therapy.

**Methods:**

Adults with migraine initiating anti-CGRP mAb monotherapy (treated, *n* = 27) and age- and sex-matched migraine controls not receiving preventive therapy (controls, *n* = 14) underwent dual-energy X-ray absorptiometry [bone mineral density (BMD) at lumbar spine, total hip, and femoral neck], lumbar trabecular bone score (TBS), and bone turnover marker assessment (serum CTX and P1NP) at baseline and 6 months. The primary endpoint was the within-treated change from baseline to month 6 in BDM and TBS. Secondary endpoint included within-patient changes in bone turnover markers and between-group change from baseline to month 6, which were assessed using linear mixed-effects models.

**Results:**

At baseline, BMD, TBS, CTX and P1NP were within the expected age-specific range in both groups. After 6 months of anti-CGRP mAb exposure all values remained within normal ranges and no clinically meaningful changes in BMD, TBS, CTX or P1NP were observed. In adjusted models, no significant group×time interactions were detected for BMD, CTX or P1NP (all *p* > 0.05). Lumbar spine TBS showed a small between-group difference in change over time (group×time interaction), with a greater decrease in treated patients than in controls (*β* = −0.052, 95% CI − 0.095 to −0.008; *p* = 0.023), with values remaining within the normal range. Anti-CGRP treatment was clinically effective, with ≥50% and ≥75% reductions in monthly migraine days achieved by 80.8 and 42.3% of patients, respectively.

**Discussion:**

This prospective, observational, controlled, cohort study suggests that anti-CGRP mAb therapy is not associated with early adverse effects on bone density, structure, or turnover, while providing substantial migraine improvement. Ongoing follow-up will determine longer-term skeletal safety.

**Clinical trial registration:**

ClinicalTrials.gov, NCT06035458.

## 1 Introduction

Migraine is a recurrent neurological disorder with a marked sex disparity, affecting women disproportionately and often requiring preventive treatment (1). In recent years, preventive treatment has been transformed by specific drugs targeting the migraine pain mediator calcitonin gene-related peptide (CGRP) or its receptor, including monoclonal antibodies (anti-CGRP mAb) and gepants. Specifically, anti-CGRP mAb display high efficacy and were overall well tolerated in pivotal trials and open-label extensions (2–9).

Beyond its established role in migraine pathophysiology, CGRP is widely expressed in several tissues, including bone. Preclinical studies indicate that CGRP is involved in the regulation of skeletal remodeling through both direct effects on bone cells and indirect neuroendocrine pathways. Experimental data suggest that CGRP may modulate osteoclast activity predominantly at early stages of differentiation, while exerting more consistent effects on osteoblast proliferation and bone formation (10, 11). In addition, CGRP signalling has been implicated in neural control of bone metabolism via interactions with leptin and sympathetic tone (12, 13). Findings from knockout models further support a role of CGRP in skeletal homeostasis, although with heterogeneous and context-dependent phenotypes (14). Together, these observations raise the hypothesis that modulation of the CGRP pathway, as it occurs with preventive anti-CGRP therapies for migraine, could negatively affect bone turnover and skeletal microarchitecture. This is especially relevant as migraine disproportionately affect women, a population already at increased risk of bone loss. Moreover, several traditional migraine preventive drugs, including anti-seizure medications and antidepressants, are known to adversely affect bone metabolism (15), and cumulative effects on bone health may occur in case of sequential or concomitant use.

Despite the widespread clinical use of anti-CGRP mAbs, human data on their effects on bone metabolism remain limited, and prospective longitudinal studies are scarce. Importantly, bone outcomes exhibit different temporal kinetics: circulating bone turnover markers [e.g., serum C-terminal telopeptide (CTX) and Procollagen Type I N-Propeptide (P1NP)] can reflect remodelling changes within weeks to months (16), whereas areal bone mineral density (BMD) measured by DXA is a later structural endpoint that requires longer observation to show detectable variation (17). Instead, trabecular bone score (TBS), as a DXA-derived surrogate of trabecular microarchitecture, may capture intermediate or early microstructural signals that can precede or occur independently of measurable BMD change (18).

Against this background, we prospectively evaluated the skeletal effects of anti-CGRP mAb therapy in patients with migraine. Using DXA-derived densitometric outcomes (including TBS) alongside serum biomarkers of bone turnover, we aimed to characterize early changes in bone quantity, microarchitecture, and metabolism compared with matched migraine controls not receiving preventive therapy. We hypothesized that 6-month exposure to anti-CGRP mAb therapy would not be associated with changes in bone mineral density, TBS or bone turnover markers. The primary objective was to characterize within-treated changes in densitometry parameters over 6 months; the secondary objective was to compare the longitudinal trajectories of densitometry parameters and bone turnover biomarkers between treated patients and controls.

## 2 Materials and methods

### 2.1 Study design and outcome

This prospective, observational, controlled, cohort study is being conducted at the Neurocenter of Southern Switzerland and is performed in accordance with the principles of the Declaration of Helsinki. The study protocol was approved by the local cantonal ethics committee in Bellinzona, Switzerland (approval date: 2022-00421CE 4049 date 31.3.2022) and the study is registered on ClinicalTrials.gov (NCT06035458, first registered 6 September 2023). The planned study duration is 24 months, with data collected at baseline (T0) and at regular 6-month intervals (T6, T12, T18, and T24). This report presents the prespecified analyses at month 6 (T6). Follow-up at months 12 and 24 is ongoing and will be reported separately. This study evaluates whether anti-CGRP mAb monotherapy over 6–24 months is associated with changes in bone density, trabecular microarchitecture, and bone turnover in patients with migraine. The primary endpoint is the within-patient change from baseline to 6 months in areal BMD at the lumbar spine (L1–L4), total hip, and femoral neck, and in lumbar spine TBS in the anti-CGRP mAb–treated group. The secondary endpoints include the within-patient change from baseline to 6 months in bone turnover markers in the anti-CGRP mAb–treated group; and the between-group differences in longitudinal changes (baseline to 6 months) in BMD, TBS, CTX, and P1NP, comparing the anti-CGRP mAb–treated group with an age- and sex-matched migraine control group not receiving preventive therapy. The primary endpoint was prespecified as within-patient change in the treated group to maximize sensitivity to short-term pharmacological effects given the small sample size, consistent with the exploratory pilot nature of this study. The control group was included to characterise background temporal variability through the secondary between-group analysis. All between-group analyses should be interpreted as exploratory.

The 6-month time point was selected based on bone biology and measurement kinetics. Bone turnover markers, including CTX and P1NP, are dynamic indicators of skeletal metabolism and typically respond within weeks to months after pharmacological modulation (16). TBS, a DXA-derived surrogate of trabecular microarchitecture, may detect early microarchitectural changes within the first 6–12 months and may precede measurable changes in areal BMD (18). In contrast, detectable changes in areal bone mineral density generally require longer observation periods (≥12 months), owing to the slow kinetics of bone mass accrual and least significant change constraints (17). Accordingly, the present 6-month assessment was designed to maximize sensitivity to early metabolic or microarchitectural bone signals potentially associated with anti-CGRP treatment, while follow-up at 12 and 24 months was planned to evaluate whether such early changes translate into sustained structural effects.

A comparator group of prospectively followed, age- and sex-matched migraine controls without preventive therapy was included to distinguish treatment-related effects from background temporal variability in densitometric, microarchitectural, and biochemical bone outcomes. Anti-CGRP mAb treatment was prescribed according to standard clinical indication and reimbursement criteria; no treatment allocation was performed by the study protocol.

### 2.2 Participants

Participants affected with migraine with or without aura referred to the Headache Out-patient Clinic at the Neurocenter of Southern Switzerland were recruited during routine evaluations between June 2022 and November 2024. The treated group included anti-CGRP mAb naïve males and females, aged 18–50 years and with migraine with or without aura (19) who were scheduled to start preventive monotherapy with an anti-CGRP mAb after failure (lack of efficacy, contraindication, or intolerance) of at least two standard preventive treatments (e.g., beta-blockers, calcium antagonists, anticonvulsants, or SSRIs) and with ≥8 monthly migraine days (MMD) across the three months preceding treatment initiation, in line with Swiss reimbursement requirements. In parallel, a control group was selected from patients with migraine who were not receiving preventive therapies and were not planning to start anti-CGRP mAb; controls were selected among migraine patients at our centre and screened to match treated participants by age and sex. No minimum MMD threshold was applied to the control group, while at least 8 monthly migraine days are required for anti CGRP monoclonals reimbursement in Switzerland. We felt it would have been unethical to preclude patients with highly frequent episodic migraine or chronic migraine from receiving appropriate preventive therapies, many of which having impact on bone metabolism. This design was intended to capture background temporal variability in bone outcomes across the migraine population as a whole. Exclusion criteria for both anti-CGRP treated and control participants included osteoporosis or prior unprovoked adult fractures; current use of medications known to affect bone metabolism (including proton pump inhibitors, SSRIs, glucocorticoids, anti-androgens/aromatase inhibitors, antiretroviral drugs, vitamin K inhibitors, or antiresorptive therapies); prior systemic glucocorticoid exposure at doses ≥5 mg prednisone-equivalent for ≥3 months; ongoing calcium or vitamin D supplementation; relevant comorbidities potentially affecting bone metabolism (e.g., diabetes, malabsorption/inflammatory bowel disease, thyroid/parathyroid dysfunction, rheumatoid arthritis, prolonged immobilization, Cushing syndrome, hypogonadism); history of drug/alcohol abuse; and, in women, menopause, pregnancy, or breastfeeding.

All participants signed a written informed consent.

### 2.3 Bone mineral measurement (bone densitometry, DXA)

Bone mineral status was evaluated through dual-energy X-ray absorptiometry (DXA; GE Lunar iDXA, GE Healthcare) scans, performed at the lumbar spine (L1–L4), total hip, and femoral neck. BMD values were expressed in absolute terms (g/cm^2^), and both T-scores and Z-scores were calculated. In line with current recommendations, Z-scores were primarily used for clinical interpretation in this premenopausal women and in men under 50 years old, while T-scores were also reported to ensure comparability with standard osteoporosis thresholds. TBS was calculated using the TBS iNsight software version 3.0.2.0 (Medimaps, Pessac, France). The same operator (C. C.) performed the DXA measurements and analyzed all scans at the end of the study to exclude inter-rater measurement variability and optimize assessment precision. Osteopenia was defined as a value for BMD more than 1 SD below the young female adult mean, but less than 2.5 SD below this value (T-score < −1 and > − 2.5 SD). Osteoporosis was defined by a value for BMD 2.5 SD or more below the young female adult mean (T-score less than or equal to −2.5 SD) or by the occurrence of a low trauma fracture (17).

### 2.4 Bone metabolism biomarkers

Laboratory assessments were performed according to standard procedures at the Laboratory of the Ospedale Regionale di Lugano (CH) and included complete blood count and routine blood chemistry [alanine aminotransferase (ALT) aspartate aminotransferase (AST), creatinine, serum protein electrophoresis, erythrocyte sedimentation rate, albumin, calcium and corrected calcium, phosphate, 25-hydroxyvitamin D (25-OH vitD), parathyroid hormone (PTH), alkaline phosphatase (ALP), and thyroid-stimulating hormone (TSH), tryptase, anti-transglutaminase antibodies, and HIV]. Bone turnover markers (CTX and P1NP) were also measured by electrochemiluminescence immunoassay (ECLIA) on fasting EDTA plasma samples.

### 2.5 Variables

At baseline, demographic and lifestyle variables (sex, age, ethnicity, weight, height, BMI, smoking status, alcohol use) were recorded. Migraine-related variables (migraine with/without aura, migraine duration, MMD, days with triptan use, days with non-triptan analgesic use, and prior preventive therapies) were collected at each time point using the headache diary/clinical records, with baseline headache burden derived from the protocol-defined pre-baseline observation period. Bone outcomes were assessed by DXA at each time point reporting areal BMD and corresponding T-scores and Z-scores, as well as lumbar spine TBS. Blood sampling for bone turnover markers was performed at each time point.

### 2.6 Statistical analysis

All analyses were performed in R (version 2025.05.0 + 496 R Foundation for Statistical Computing, Vienna, Austria), using standard packages. Missing data were not imputed; analyses used complete-case observations for each specific comparison or model. All tests were two-sided, and *p* values < 0.05 were considered statistically significant.

As no prospective data were available on the effect of 6 months of anti-CGRP mAb therapy on densitometry outcomes, the planned sample size was based on the primary within-treated comparison (T0 vs. T6). A target of 28 treated participants was estimated to provide 80% power to detect a medium effect size (0.5) at a two-sided *α* = 0.05, allowing for ~15% attrition. Of note, the sample size was calculated for the primary within-treated comparison and was not designed to achieve specific statistical power for between-group analyses; accordingly, all between-group comparisons should be interpreted as exploratory. A comparator group of 14 age- and sex-matched migraine controls without preventive therapy was included for reference.

Continuous variables were reported as median and interquartile range (IQR). Categorical variables were reported as counts (%). Baseline between-group comparisons used the Wilcoxon rank-sum test for continuous variables and the χ^2^ test or Fisher’s exact test for categorical variables, as appropriate. Within the treated group, changes from baseline (T0) to 6 months (T6) were assessed using paired non-parametric tests (Wilcoxon signed-rank test for continuous outcomes; McNemar test for binary outcomes) and visualized using paired trajectory plots and change-score (ΔT6–T0) distributions. Longitudinal changes from T0 to T6 were evaluated using linear mixed-effects models with a subject-specific random intercept, including fixed effects for group, time, and the group×time interaction. For each outcome, both unadjusted models (group, time, group×time only) and adjusted models were fitted.

Prespecified covariate adjustment followed outcome-specific rules: BMD, T-scores and bone turnover markers were adjusted for age, sex, baseline BMI, smoking status, and baseline 25-OH vit D. For Z-score outcomes, age and sex were not included as covariates, as these factors are already mathematically incorporated in the Z-score normalization (i.e., Z-scores are age- and sex-adjusted by construction). TBS was adjusted for age, sex, smoking status, and baseline 25-OH vit D.

Given the exploratory, hypothesis-generating nature of this pilot study and the multiplicity of outcomes assessed, all *p*-values are reported nominally without correction for multiple comparisons. Results should be interpreted as signal-detection estimates. Statistical analyses followed the approach prespecified in the study protocol.

## 3 Results

### 3.1 Baseline

#### 3.1.1 Demographics and headache characteristics

In total, 42 patients were enrolled: 28 in the anti-CGRP mAb treated group and 14 in the control group. One treated participant withdrew consent after the baseline visit for personal reasons, leaving 41 participants for the analyses. All individuals were Caucasian, 87.8% were female, median age was 37 years (IQR 31–44) median BMI was 21.9 kg/m^2^ (IQR 20–25.2). Overall, baseline characteristics were similar between groups except for current smoking, which was more frequent in the anti-CGRP mAb-treated [8/41 (19.5%) patients] than in control [0/14 (0%) patients] group (*p* = 0.035) (Table 1).

**Table 1:** Baseline characteristics of the study cohort by treatment group.

Characteristic	Overall *N* = 41	Control *N* = 14	Treated *N* = 27	*p*-value
Sex				
Female *n* (%)	36 (87.8%)	13 (92.86%)	23.00 (85.19%)	0.645
Male *n* (%)	5 (12.2%)	1 (7.14%)	4 (14.81%)	
Age (year),	37.00 [31.00, 44.00]	37.50 [32.00, 39.00]	37.00 [29.00, 45.00]	0.826
Caucasian	41 (100%)	14 (100%)	27 (100%)	
Weight (kg),	63.00 [55.00, 70.00]	58.50 [55.00, 63.00]	64.00 [55.00, 76.00]	0.289
High (cm),	167.00 [160.00, 173.00]	169.50 [163.00, 174.00]	166.00 [159.00, 172.00]	0.277
BMI (kg/m^2^),	21.90 [20.00, 25.20]	21.10 [19.30, 23.10]	22.50 [20.60, 29.40]	0.105
Smoker (yes), *n* (%)	8 (19.51%)	0	8 (29.63%)	**0.035**
Alcohol (yes), *n* (%)	5 (12.20%)	2(14.29%)	3 (11.11%)	>0.999
Aura (yes), *n* (%)	12 (29.27%)	5 (35.71%)	7 (25.93%)	0.719
Migraine duration (year),	15.00 [5.50, 21.00]	10.00 [5.00, 20.00]	17.50 [10.00, 22.00]	0.205
Monthly migraine days month before baseline,	8.00 [5.00, 12.00]	2.50 [2.00, 5.00]	10.00 [8.00, 20.00]	**<0.001**
Days with triptan use, 1 month before baseline,	2.00 [0.00, 8.00]	0	8.00 [2.00, 10.00]	**<0.001**
Days with analgesic (no triptan) use, 1 month before baseline,	3.00 [0.00, 7.00]	2.50 [2.00, 3.00]	4.00 [0.00, 9.00]	0.718
Previous preventive treatment				
Anti epileptic agents (yes), *n* (%)	20 (48.8%)	0	20 (74.1%)	< 0.001
Antidepressant (yes), *n* (%)	16 (39.0%)	0	16 (59.3%)	< 0.001
Beta blockers (yes), *n* (%)	9 (22.0%)	0	9 (33.3%)	0.017
Anti-CRGP mAb started				
Erenumab (yes), *n* (%)	5 (12.20%)	0	5 (18.52%)	0.146
Galcanezumab (yes), *n* (%)	8 (19.51%)	0	8 (29.63%)	**0.035**
Fremanezumab (yes), *n* (%)	8 (19.51%)	0	8 (29.63%)	**0.035**
Eptinezumab (yes), *n* (%)	6 (14.63%)	0	6 (22.22%)	0.079

Demographic and migraine-related variables are reported for the overall cohort and stratified by anti-CGRP mAb treatment status. Categorical variables are presented as n (%), and continuous variables as median [interquartile range]. *p*-values are from two-sided between-group comparisons (χ^2^ test or Fisher’s exact test for categorical variables; Wilcoxon rank-sum test for continuous variables) comparing anti-CGRP mAb–treated patients with controls. *p*-value ≤ 0.05 is marked in bold.

As expected, baseline headache frequency was higher in the anti-CGRP mAb treated group [median MMD 10 days (IQR 8–20)] than in controls [2.5 days (IQR 2–5); (*p* < 0.001)]. Similarly, median days of triptan use were greater among anti-CGRP mAb treated patients [8 (IQR 2–10) vs. 0 (IQR 0–0); (*p* < 0.001)].

The most frequently used anti-CGRP mAbs were galcanezumab and fremanezumab (each 29.6%).

#### 3.1.2 Bone density and microarchitecture (DXA and TBS)

Baseline DXA measurements and TBS were within the expected range for age in both groups. Median lumbar spine BMD was 1.15 g/cm^2^ (IQR 1.09–1.24), total hip BMD 0.96 g/cm^2^ (IQR 0.91–1) and femoral neck BMD 0.93 g/cm^2^ (0.87–0.99), without between-group differences. Corresponding median Z-scores were −0.6 [−0.90, 0.30] at the femoral neck and −0.3 at the lumbar spine [−0.80, 0.60] and hip [−0.90, 0.10], consistent with normal bone status. The median lumbar TBS was 1.47 (IQR 1.41–1.56), with no differences between groups (Table 2).

**Table 2:** Baseline bone mineral density (BMD) and lumbar trabecular bone score (TBS) in the overall cohort and by treatment group.

Characteristic	*N*	Overall	Control	Treated	*p*-value
BMD lumbar L1–L4 g/cm^2^	41	1.15 [1.09, 1.24]	1.15 [1.06, 1.26]	1.16 [1.09, 1.23]	0.773
T-score lumbar L1-L4	38	−0.30 [−1.00, 0.60]	−0.30 [−1.00, 0.60]	−0.30 [−0.85, 0.45]	0.683
Z-score lumbar L1–L4	41	−0.30 [−0.80, 0.60]	−0.10 [−0.60, 1.00]	−0.30 [−1.00, 0.50]	0.248
BMD total hip g/cm^2^	41	0.96 [0.91, 1.00]	0.98 [0.87, 1.04]	0.96 [0.91, 1.00]	0.670
T-score total hip	38	−0.45 [−1.10, 0.10]	−0.20 [−1.10, 0.30]	−0.65 [−0.90, 0.05]	0.606
Z-score total hip	41	−0.30 [−0.90, 0.10]	−0.05 [−0.90, 0.60]	−0.30 [−1.00, 0.10]	0.259
BMD femoral neck g/cm^2^	41	0.93 [0.87, 0.99]	0.97 [0.85, 0.99]	0.91 [0.87, 0.99]	0.518
T-score femoral neck	38	−0.65 [−1.10, 0.00]	−0.25 [−1.10, 0.10]	−0.70 [−1.20, −0.35]	0.325
Z-score femoral neck	41	−0.60 [−0.90, 0.30]	0.05 [−0.90, 0.40]	−0.60 [−0.90, 0.20]	0.321
TBS lumbar (L1–L4)	41	1.47 [1.41, 1.56]	1.45 [1.40, 1.47]	1.51 [1.42, 1.59]	0.086

Values are reported as median [interquartile range]. *p*-values are from two-sided Wilcoxon rank-sum tests comparing anti-CGRP mAb–treated patients with controls.

#### 3.1.3 Bone turnover markers and biochemical parameters

At baseline, bone turnover markers and biochemical parameters were within normal reference ranges. Median concentration of CTX was 0.36 μg/L (IQR 0.25–0.57), while median P1NP and ALP concentrations were 44.20 μg/L (36.10–67.20) and 53 U/L (44–63), respectively. ALP levels were slightly higher in the treated group compared with controls (61.0 vs. 47.6 U/L; *p* = 0.023), while median 25-OH Vit D values were slightly higher in controls than treated patients [30.90 (IQR 2.30–40.80) vs. 27.50 (IQR 17.40–29.10) ng/mL; (*p* = 0.041)]. Other biochemical parameters - including total and corrected calcium, phosphate, PTH, and creatinine - were comparable between groups (Table 3).

**Table 3:** Baseline bone turnover markers and biochemical parameters in the overall cohort and by treatment group.

Characteristic	Overall *N* = 41	Control *N* = 14	Treated *N* = 27	*p*-value
CTX μg/L	0.36 [0.25, 0.57]	0.31 [0.23, 0.39]	0.42 [0.25, 0.57]	0.221
P1NP μg/L	44.20 [36.10, 67.20]	43.00 [33.30, 55.90]	48.10 [36.10, 69.30]	0.336
ALP U/L	53.00 [44.00, 63.00]	46.50 [38.00, 53.00]	54.00 [48.00, 68.00]	**0.023**
25-OH VitD ng/mL	26.50 [19.80, 31.40]	30.90 [24.30, 40.80]	25.70 [17.40, 29.10]	**0.041**
Calcium mmol/L	2.29 [2.24, 2.33]	2.29 [2.24, 2.34]	2.29 [2.24, 2.33]	0.923
Corrected calcium mmol/L	2.19 [2.13, 2.23]	2.18 [2.12, 2.23]	2.20 [2.14, 2.24]	0.37
Creatinine μmol/L	71.00 [66.00, 82.00]	73.50 [61.00, 79.00]	71.00 [68.00, 83.00]	0.509
Phosphate mmol/L	1.06 [0.95, 1.20]	1.11 [0.92, 1.20]	1.03 [0.95, 1.21]	0.752
PTH pmol/L	5.05 [3.70, 5.90]	4.85 [4.00, 5.90]	5.25 [3.15, 5.90]	0.683

Values are reported as median [interquartile range]. *p*-values are from two-sided Wilcoxon rank-sum tests comparing anti-CGRP mAb–treated patients with controls. CTX, C-terminal telopeptide of type I collagen; P1NP, procollagen type I N-terminal propeptide; ALP, Alkaline phosphatase, 25-OH VitD, 25-hydroxyvitamin D; PTH, Parathyroid hormone. *p*-value ≤ 0.05 is marked in bold.

### 3.2 Within anti-CGRP treated group analysis at 6 months

#### 3.2.1 Bone density, microarchitecture and bone turnover markers

**Figure 1:**
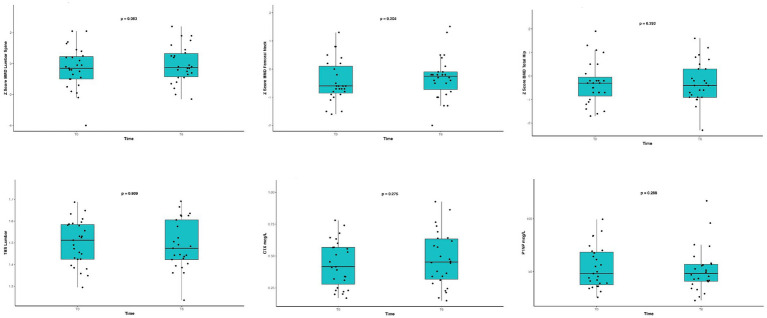
For all plots, boxplots show the median and interquartile range (IQR). Individual observations are displayed as jittered points. *p*-values indicate within-group comparisons between T0 and T6.

In the anti-CGRP treated group, BMD and TBS values at 6 months remained within the normal range, with no differences *versus* baseline. Similarly, at month 6 bone turnover markers and biochemical parameters remained all in the normal range, with small increases observed in total calcium (+0.04 mmol/L, ~ + 2%) and PTH (+0.62 pmol/L, ~ + 13%; both *p* < 0.05) (Table 4; Figure 1)

**Table 4:** Within-patient changes from baseline (T0) to 6 months (T6) in anti-CGRP mAb–treated patients.

Outcome	*N*	Baseline (T0)	Month 6 (T6)	*p*-value
Bone densitometry				
BMD Lumbar L1–L4 g/cm^2^	26	1.15 [1.09; 1.23]	1.16 [1.11; 1.25]	0.374
T-score Lumbar L1-L4	23	−0.40 [−0.85; 0.45]	−0.40 [−0.80; 0.45]	0.741
Z-score colonna L1–L4	26	−0.35 [−1.00; 0.47]	−0.25 [−0.85; 0.65]	0.083
BMD toal hip g/cm^2^	25	0.96 [0.91; 1.00]	0.95 [0.92; 1.02]	0.696
T-score total hip	23	−0.70 [−0.90; 0.05]	−0.40 [−0.85; 0.20]	0.499
Z-score total hip	25	−0.30 [−1.00; 0.10]	−0.40 [−0.90; 0.30]	0.293
BMD femoral neck g/cm^2^	25	0.91 [0.87; 0.99]	0.94 [0.89; 0.97]	0.989
T-score femoral neck	23	−0.70 [−1.20; −0.35]	−0.60 [−1.15; −0.15]	0.881
Z-score femorl neck	26	−0.60 [−0.88; 0.15]	−0.25 [−0.73; −0.10]	0.204
TBS lumbar (L1–L4)	25	1.49 [1.42; 1.58]	1.48 [1.42; 1.60]	0.809
Bone turnover biomarkers				
CTX μg/L	26	0.43 [0.31; 0.57]	0.45 [0.32; 0.63]	0.275
P1NP μg/L	26	48.95 [36.78; 68.88]	48.15 [40.73; 56.83]	0.288
ALP U/L	26	53.50 [48.25; 65.25]	56.00 [52.50; 69.00]	0.067
25-OH VitD ng/mL	26	25.75 [19.15; 29.00]	21.25 [17.95; 30.78]	0.611
Calcium mmol/L	26	2.29 [2.24; 2.33]	2.31 [2.27; 2.36]	**0.030**
Corrected calcium mmol/L	23	2.19 [2.13; 2.23]	2.21 [2.15; 2.25]	0.135
Creatinine μmol/L	26	71.50 [68.25; 82.75]	75.00 [68.00; 80.00]	0.855
Phosphate mmol/L	26	1.03 [0.95; 1.17]	1.10 [1.02; 1.19]	0.587
PTH pmol/L	23	5.40 [3.50; 5.90]	5.50 [3.95; 7.00]	**0.014**

Bone mineral density (BMD), densitometric T- and Z-scores, lumbar trabecular bone score (TBS), bone turnover biomarkers reported as median [p25–p75] (n as indicated). *p*-values are from two-sided paired Wilcoxon signed-rank tests. Normal range are reported in Supplementary material. CTX, C-terminal telopeptide of type I collagen; P1NP, procollagen type I N-terminal propeptide; ALP, Alkaline phosphatase, PTH, parathyroid hormone. *p*-value ≤ 0.05 is marked in bold.

#### 3.2.2 Headache frequency and medication use

Among patients treated with anti-CGRP mAbs a marked clinical improvement was observed over the 6-month follow-up. The mean number of MMD decreased from 13.0 ± 7.1 to 4.6 ± 5.2 (*p* < 0.001), corresponding to a median reduction of 7 days per month. Days of triptan use declined from 6.0 ± 4.5 to 1.9 ± 2.6 (*p* = 0.002), and the number of days using non-triptan analgesics decreased from 5.7 ± 6.5 to 1.4 ± 1.9 (*p* = 0.001). Clinically meaningful responder thresholds were frequently achieved (96.2%). At month 6, 80.8% of treated patients reached a ≥ 50% reduction in MMD, and 42.3% achieved a ≥ 75% reduction.

### 3.3 Longitudinal comparison between groups

**Figure 2:**
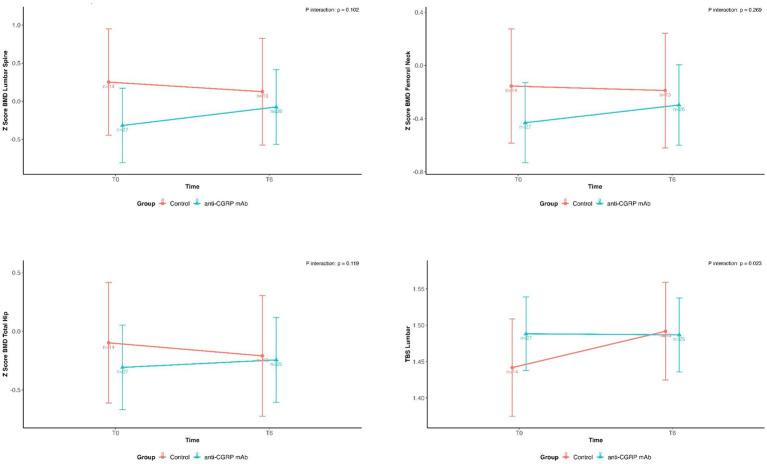
Adjusted trajectories of densitometric outcomes and trabecular microarchitecture from baseline (T0) to 6 months (T6) by study group. Panels show model-based estimated marginal means (EMMs) for BMD *Z*-scores at the lumbar spine, total hip, and femoral neck, and for lumbar trabecular bone score (TBS) in anti-CGRP mAb-treated patients (blue lines) and controls (red lines). *Z*-score models were adjusted for BMI, smoking status, and vitamin D; TBS were adjusted for age, sex, smoking status, and 25-OH vit D. Error bars represent 95% confidence intervals. Numbers next to each estimate indicate the sample size with available data at the corresponding time point. The *p*-value is reported for each outcome.

In the adjusted longitudinal mixed-effects models, most densitometry outcomes were stable between baseline and month 6 with no evidence of differential change between the two groups. Specifically, no significant group×time interactions were observed for lumbar spine, total hip, or femoral neck BMD, nor for the corresponding T-scores and Z-scores (all p for interaction > 0.05). In contrast, TBS showed a positive group×time interaction, consistent with a small relative decrease from baseline to month 6 in the anti-CGRP group versus controls (*β* = −0.052, 95% CI − 0.095 to −0.008; *p* = 0.023); observed values remained broadly within the normal range (Table 5; Figure 2).

**Table 5:** Adjusted longitudinal mixed-effects models for densitometry outcomes and bone turnover markers (baseline to 6 months).

Outcome	*N*	β	95% CI	*p* value
Bone densitometry				
BMD Lumbar L1–L4 g/cm^2^	41	0.038	[−0.041; 0.117]	0.34
T-score Lumbar L1-L4	39	0.132	[−0.035; 0.300]	0.117
Z-score colonna L1–L4	41	0.369	[−0.077; 0.814]	0.102
BMD toal hip g/cm^2^	41	0.009	[−0.018; 0.036]	0.502
T-score total hip	39	0.107	[−0.133; 0.347]	0.372
Z-score total hip	39	0.175	[−0.047; 0.398]	0.95
BMD femoral neck g/cm^2^	41	0.001	[−0.032; 0.034]	0.809
T-score femoral neck	41	0.034	[−0.251; 0.320]	0.269
Z-score femorl neck	39	0.168	[−0.135; 0.470]	0.268
TBS lumbar (L1–L4)	41	−0.052	[−0.095; −0.008]	**0.023**
Bone turnover markers				
CTX μg/L	41	0.042	[−0.062; 0.146]	0.415
P1NP μg/L	41	0.908	[−7.899; 9.714]	0.836
ALP U/L	41	5.010	[−2.546; 12.565]	0.187
25-OH Vit D ng/mL	41	6.190	[−1.704; 14.085]	0.122
Calcium mmol/L	41	−0.002	[−0.060; 0.056]	0.946
Corrected calcium mmol/L	41	−0.032	[−0.091; 0.026]	0.264
Creatinine μmol/L	41	−0.400	[−6.028; 5.228]	0.886
Phosphate mmol/L	41	−0.065	[−0.160; 0.031]	0.177
PTH pmol/L	41	0.412	[−0.613; 1.437]	0.42

Models included fixed effects for group, time, and the group×time interaction, and were adjusted for prespecified covariates as follows: BMD, T-scores and bone markers turnover (age, sex, baseline BMI, smoking status, and 25-OH vitamin D); Z-scores (baseline BMI, smoking status, and 25-OH vitamin D); TBS (age, sex, smoking status, and 25-OH vitamin D). β represents the adjusted between-group difference in change from baseline to month 6 derived from mixed-effects models.CTX, C-terminal telopeptide of type I collagen; P1NP, procollagen type I N-terminal propeptide; ALP, alkaline phosphatase; PTH, parathyroid hormone. *p*-value ≤ 0.05 is marked in bold.

**Figure 3:**
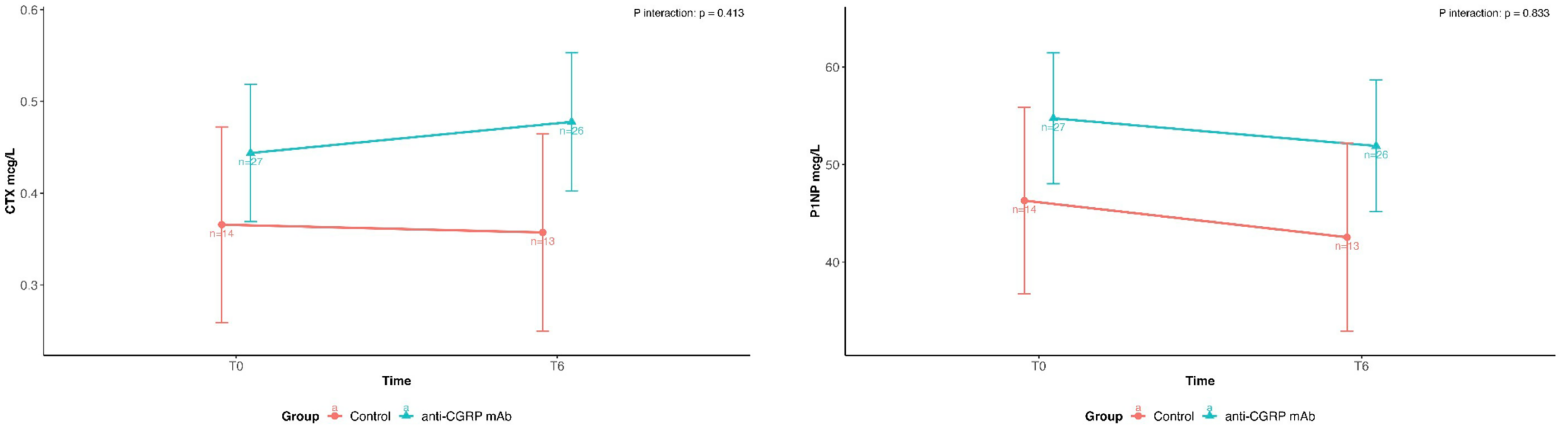
Adjusted trajectories of bone turnover markers from baseline (T0) to 6 months (T6) by study group. Panels show model-based estimated marginal means (EMMs) for serum CTX and P1NP in anti-CGRP mAb-treated patients (blue lines) and controls (red lines), derived from linear mixed-effects models including the Group×Time interaction and prespecified covariates (age, sex, body mass index (BMI), smoking status, and baseline 25 OH vit D). Error bars represent 95% confidence intervals. Numbers next to each estimate indicate the sample size with available data at the corresponding time point. The *p*-value is reported for each outcome.

For bone turnover markers and biochemical parameters, there was no evidence of changes over time for CTX, P1NP, and ALP (all p for group × time interaction ≥ 0.05). Among laboratory measures, serum calcium increased slightly from baseline to month 6 (main effect of time, *p* = 0.007), with a comparable change in both groups (no group×time interaction, *p* = 0.946). The remaining markers showed no evidence of differential trajectories between groups (Table 5; Figure 3).

Unadjusted longitudinal models are reported separately (Supplementary material).

## 4 Discussion

This study evaluated changes in bone health markers—encompassing densitometric measures (DXA-derived BMD, T- and Z-scores, and lumbar spine TBS) and circulating bone turnover and biochemical parameters—in patients with migraine treated with anti-CGRP mAbs over 6 months, compared with age- and sex-matched controls.

At baseline, all structural and biochemical measures of bone health were within the expected age-specific range in both groups. Over 6 months, no clinically meaningful deterioration in BMD, TBS or bone turnover markers was observed in treated patients, and most outcomes did not differ in their temporal evolution between groups. At the same time, anti-CGRP therapy was associated with the expected improvement in migraine outcomes over 6 months. Mean MMD decreased by ~65%, with parallel reductions in triptan-use days (~68%) and non-triptan analgesic-use days (~75%). More than 80% of patients achieved a ≥ 50% reduction in MMD, and over 40% reached a ≥ 75% reduction.

Human evidence regarding the skeletal impact of CGRP pathway inhibition remains limited, and prospective longitudinal data are scarce. Bone turnover markers (CTX and P1NP) are early readouts of remodeling activity and may change within weeks to months (16), whereas areal BMD is a late structural endpoint that typically requires ≥12 months to show detectable variation (17). TBS lies between these domains and may capture early microarchitectural changes within the first 6–12 months (18). Our findings support the interpretation that short-term exposure to anti-CGRP monoclonal antibodies is not associated with overt adverse effects on bone density or with a consistent shift in bone turnover markers. Similarly, lumbar spine TBS remained within the expected range at both baseline and 6 months, and absolute changes were small. We nevertheless observed a modest group×time interaction for TBS (*β* = −0.052; *p* = 0.023), a finding that warrants careful contextualization. The estimated follow-up value of 1.46 remains well above pathological thresholds (1.35 and 1.20) (18) and the 0.052-unit change falls below the published LSC for TBS (0.06–0.11) (20), likely placing it within measurement noise. As a secondary exploratory outcome, this nominal *p*-value may reflect a false positive, particularly given relevant confounders including significantly higher smoking prevalence in the treated group (19.5% vs. 0%, *p* = 0.035) and baseline differences in ALP, vitamin D, and migraine burden. However, this signal warrants further investigation, including its longer follow up already planned in this 2-year study. By incorporating a longitudinal design with a comprehensive panel of bone turnover markers and a control group, these prospective data extend our earlier cross-sectional study in migraine patients treated with anti-CGRP monoclonal antibodies, in which no association was observed between treatment duration (mean exposure of 15 months) and BMD or TBS, nor an excess prevalence of osteopenia or osteoporosis compared with age- and sex-expected values (21). In another study by Ray and colleagues the bone formation marker P1NP at an early follow-up significantly increased approximately 3 months after treatment initiation in 45 patients receiving CGRP (22). While the magnitude of the reported P1NP increase might be greater than what is typically attributed to known biological variability (23), the absolute values remained largely within established reference ranges, similar to our findings. Moreover, the increase in bone formation marker P1NP might also reflect the discontinuation of specific prior preventive migraine therapies with known osteocatabolic effect (15, 21, 22). Unfortunately, the authors do not provide this information in their paper preventing us from meaningful comparisons with our study population.

A recently presented, prospective uncontrolled study in patients with chronic or high-frequency episodic migraine treated with anti-CGRP mAb for one year reported a small but statistically significant decline in lumbar spine BMD, in women aged ≥50 years, while bone turnover markers remained largely unchanged (24). In a separate report from the same group, a cohort of women with chronic migraine, eligible for anti-CGRP therapy,already exhibited lower BMD, TBS, and vitamin D levels compared with healthy controls prior to treatment initiation, suggesting that migraine burden and/or associated lifestyle factors may contribute to baseline skeletal vulnerability (24), and highlighting the importance to evaluate the bone impact of anti CGRP therapies in a controlled setting. Actually, emerging evidence suggests that migraine itself may be linked to altered bone metabolism independently of anti-CGRP therapy. In a case series of 19 migraine patients—predominantly premenopausal women, similar to our cohort—Lerario reported uniformly elevated CTX concentrations, indicating increased bone resorption independently of specific preventive treatments (25). In addition, population-based data have shown an increased risk of migraine among individuals with osteoporosis, suggesting shared pathophysiological mechanisms or risk factors (15, 26).

This study has several strengths. First, it is the first prospective, observational, controlled, cohort study investigating bone health markers including both structural (DXA and TBS) and dynamic (bone markers turnover) before and during anti-CGRP mAb therapy. Importantly, the inclusion of a prospectively followed, age- and sex-matched migraine control group without preventive therapy likely reduced the risk that observed changes reflected background temporal variability rather than treatment exposure. Second, multidimensional bone measurements were performed in a single centre using standardized protocols, limiting measurement heterogeneity. Third, our within-patient design in the treated group increased sensitivity to detect short-term changes in bone density and metabolism.

Limitations should also be acknowledged. This report covers a limited follow up period. Despite this, the time window is appropriate for bone dynamic readouts (CTX and P1NP) and for potential early microarchitectural signals on TBS, whereas definitive structural changes in areal BMD typically require longer observation and will be studied over an appropriate long term analysis. Second, the relatively small sample size was calculated for the primary within-treated comparison, and not for between-group analyses. In addition, the 2:1 allocation ratio further reduces between-group power. Overall, this precludes detection of small size effects or rare clinical outcomes. Accordingly, results should be interpreted as signal-detection and estimation rather than definitive evidence of long-term bone safety, and should primarily inform the design of larger confirmatory studies. Third, the study was monocentric and included mainly of relatively young, premenopausal Caucasian women, limiting generalizability. However, it also represents a strength for this exploratory phase, as it minimizes in particular confounding from age-related bone loss and postmenopausal osteoporosis. Moreover, we could not provide separate analysis for the different monoclonal antibodies due to the small sample size. Of note, receptor blockade by erenumab may more completely abolish CGRP-mediated osteoblast anabolic signaling than ligand-targeting agents (fremanezumab, galcanezumab, eptinezumab), which leave endogenous CGRP able to bind alternative subtypes (AMY1R, AMY2R) expressed in bone; whether this theoretical difference is clinically meaningful requires investigation in larger cohorts (27). We did not systematically collect data on physical activity, dietary and sun exposure all of which may influence bone metabolism. In particular, MMD reduction and responder rate in treated patients may have been accompanied by increased physical activity, representing a potential “masking effect” that could offset any osteocatabolic CGRP inhibition and bias results toward the null.

In addition, at baseline, the treated group differed significantly from controls in smoking prevalence, headache burden, and use of concomitant preventive medications, reflecting a more severe migraine phenotype. While these imbalances could theoretically contribute to differential changes, they would be expected to bias results toward worse bone outcomes in the treated group.

For clinicians, these 6-month data provide practical reassurance that, in a relatively young cohort, anti CGRP mAb treatment was not accompanied by clinically evident short-term harm to bone density or structure, or a bone turnover profile suggestive of accelerated bone loss. Treatment duration remains a critical variable in safety assessment; as recently reviewed, questions remain regarding long-term and non-specific biological effects of sustained CGRP pathway inhibition in tissues where CGRP plays a homeostatic role (28). Ongoing 12- and 24-month follow-up assessments are planned and will be reported separately.

## Data Availability

The raw data supporting the conclusions of this article will be made available by the authors, without undue reservation.
