# Supplementary figures and images for: Anti-CGRP monoclonal antibody therapy for migraine is not associated with early adverse bone effects: a prospective, observational, controlled, cohort study
Source: Front Neurol. 2026 May 29;17:1851653. doi: 10.3389/fneur.2026.1851653 (PMC13267814; doi:10.3389/fneur.2026.1851653)

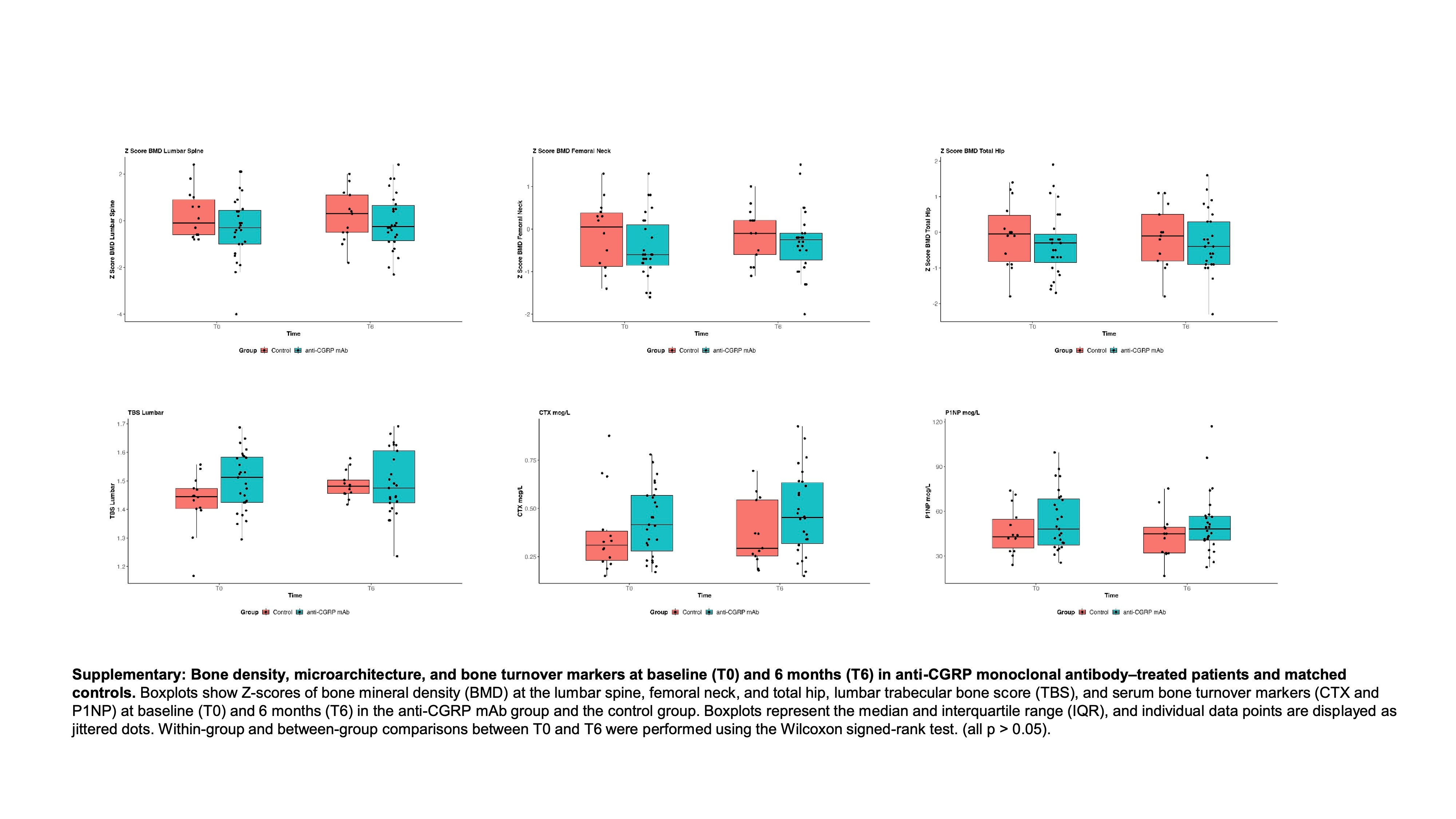

Supplement: Supplementary file 1 [file Image_1.JPEG]
